# Effects of acupuncture on musculoskeletal pain: an evidence map

**DOI:** 10.3389/fmed.2025.1575226

**Published:** 2025-08-11

**Authors:** Lin Ang, Eunhye Song, Tae-Young Choi, Ji Hee Jun, Boram Lee, Mi Hong Yim, Hye Won Lee, Myeong Soo Lee

**Affiliations:** ^1^Korean Medicine Science Research Division, Korea Institute of Oriental Medicine, Daejeon, Republic of Korea; ^2^Global Cooperation Center, Korea Institute of Oriental Medicine, Daejeon, Republic of Korea; ^3^Digital Health Research Division, Korea Institute of Oriental Medicine, Daejeon, Republic of Korea; ^4^Korean Medicine Convergence Research Division, Korea Institute of Oriental Medicine, Daejeon, Republic of Korea

**Keywords:** evidence map, systematic review, overview, acupuncture, musculoskeletal pain

## Abstract

**Background:**

Musculoskeletal pain is a leading cause of disability and reduced quality of life worldwide. Given the growing interest in complementary and alternative therapies, acupuncture has been widely explored as a potential treatment for alleviating musculoskeletal pain. This evidence map aimed to identify, describe, and summarize the current available evidence about acupuncture interventions on musculoskeletal pain.

**Methods:**

For this map, searches were conducted in PubMed, Embase, Cochrane Library, Allied and Complementary Medicine Database (AMED), Web of Science, and Epistemonikos to identify systematic reviews (SRs) with meta-analysis published up to 23 August 2024. Included SRs were independently assessed for eligibility in pairs. The data from the eligible SRs were extracted and evaluated for methodological quality using AMSTAR 2. The findings were tabulated and mapped using bubble plots.

**Results:**

A total of 111 SRs fulfilled the eligibility criteria and were included in this evidence map. All of the SRs included manual acupuncture or electroacupuncture. Comparators included in SRs involved active comparators, inactive comparators, sham acupuncture, and no intervention. The included 111 SRs were categorized into 35 musculoskeletal pain conditions. The short-term effects of acupuncture showed a positive effect across most comparators in major musculoskeletal pain. All included SRs were rated low or critically low in terms of methodological quality.

**Conclusion:**

This evidence map demonstrated that acupuncture has favorable effects on major musculoskeletal disorders. Further improvements in the quality of evidence should be prioritized and more clinical trials on the acupuncture for treating musculoskeletal pain are needed.

## 1 Introduction

Musculoskeletal pain is the most common symptom associated with musculoskeletal disorders affecting the bones, muscles, joints, ligaments, and tendons. This type of pain can be acute and chronic and may be localized to one area or widespread throughout the body (1). According to the Global Burden of Disease (GBD) analysis, approximately 1.71 billion individuals worldwide are living with musculoskeletal conditions (2). Musculoskeletal pain can arise from various causes, including injuries, degenerative conditions, and poor posture. Diagnosing musculoskeletal pain can be complicated and requires a thorough examination which involves reviewing medical history and performing physical examinations. Additional radiology tests such as X-rays, CT scans, and MRI may also be needed to identify the specific cause of pain (3). At present, musculoskeletal pain is treated with both pharmaceutical and non-pharmacological approaches, depending on the underlying cause. Most guidelines for the management of musculoskeletal pain mainly focus on medications, physical therapy, manual therapies, as well as lifestyle modifications (4).

Acupuncture is a form of traditional medicine involving the insertion of needles into specific points of the body to relieve various symptoms and stimulate holistic healing. Acupuncture therapy, whether used alone or in combination with other therapies like physiotherapy, has gained widespread use for treating musculoskeletal pain in many countries around the world (3). The number of published studies on this topic has been steadily increasing over the past two decades, indicating growing interest and research in this field (5, 6). A comprehensive review of alternative therapies, including acupuncture, for chronic pain management highlighted that acupuncture is a promising method for reducing chronic musculoskeletal pain (7).

An evidence map is a form of review that evaluates a broad subject to identify the current state of the evidence, knowledge gaps, and areas in need of future research, and that visualizes the findings in an easily comprehensible format (8, 9). As new evidence continues to accumulate, evidence of the effectiveness of acupuncture on musculoskeletal pain should be further explored and analyzed. Therefore, this evidence map is to identify, describe, and summarize the current available evidence about acupuncture interventions on musculoskeletal pain.

## 2 Methods

This evidence map was performed based on the methodology proposed by the Global Evidence Mapping (GEM) (10) and was reported according to the Preferred Reporting Items for Systematic Reviews and Meta-Analyses–Scoping Review extension (PRISMA-ScR) (11) and Preferred Reporting Items for Overviews of Reviews including harms checklist (PRIO-harms) (12). An evidence map aims to identify, classify, and describe patterns, trends, or gaps to facilitate a more in-depth thematic understanding of prior research on a given topic (8, 9).

### 2.1 Literature search

Six databases were searched from inception to 18 April 2024, including PubMed, Embase, Cochrane Library, Allied and Complementary Medicine Database (AMED), Web of Science, and Epistemonikos. Searches for all databases were rerun and updated until 23 August 2024, before data synthesis to ensure we included newly published records. The search terms of keywords, subject headings, and index terms describing acupuncture and musculoskeletal pain, such as “acupuncture,” “electroacupuncture,” “musculoskeletal pain,” “carpal tunnel syndrome,” “fibromyalgia,” “low back pain,” and “osteoarthritis” among others, were included. The detailed search strategy for the PubMed database is presented in Supplementary Table 1.

### 2.2 Study selection

Following the search, all the identified records were uploaded to Endnote reference management software, and duplicates were removed. Two reviewers (LA and ES) independently screened the title and abstract for relevance. Full texts of the potential included systematic reviews (SRs) were then reviewed in duplicate, with any discrepancies resolved by the other reviewers (T-YC and JJ).

Studies were included if they met the following criteria: (a) participants with any musculoskeletal pain; (b) included invasive acupuncture as intervention (manual acupuncture, electro-acupuncture, and warm acupuncture); (c) where acupuncture intervention was compared with controls such as sham or placebo acupuncture, no treatment, or another intervention (pharmacological and non-pharmacological); (d) assessed at least one pain outcome measured by validated scales such as visual analog scale (VAS) or numeric rating scale (NRS); and (e) published SRs with meta-analysis. Systematic reviews that covered other interventions were still considered if results for acupuncture were reported separately. Network meta-analysis reviews were also eligible if results for meta-analysis of pain outcomes were reported separately.

Exclusion criteria for this map included (a) studies focused on general chronic or acute pain; (b) only healthy participants; (c) studies involving non-invasive acupuncture as one of the interventions (acupressure, laser acupuncture, or auriculotherapy), dry needling – acupuncture at a local point instead of standard acupoints, or additional elements such as bee venom, pharmacopuncture; (d) studies involving non-acupuncture-related interventions (cupping, herbal medicine, etc.); (e) studies that did not measure pain outcome; (f) SRs without meta-analysis; and (g) preprints and conference abstracts. Reasons for the exclusion of potential included SRs were recorded and reported, with any discrepancies resolved by additional reviewers (T-YC and JJ).

### 2.3 Data collection

Data extraction was performed by two independent reviewers in pairs (T-YC and JJ). All data extracted were further checked by another reviewer (ES). Data extracted included intervention characteristics, type of musculoskeletal disorders, comparators types, number of articles in a review, and impact of intervention. For acupuncture interventions, the acupuncture types were extracted based on the original authors’ report. When SRs that included specific acupuncture such as manual acupuncture, electro-acupuncture, and warm acupuncture, we report the type of acupuncture as reported by the authors. When the SRs are reported simply as acupuncture as they included several types of acupunctures, we did not reclassify and referred to them as acupuncture as well. The impact of the intervention is reported in accordance with the findings of the meta-analysis.

### 2.4 Quality assessment

The quality of the included SRs was evaluated by two independent reviewers (LA and BL) using the AMSTAR 2 tool (13), with any discrepancies resolved by the other reviewer (ML). AMSTAR 2 contains 16 items with 7 key items. The overall quality of SRs is divided into four levels: high – no or one non-key item unavailable; moderate – more than one non-key item unavailable; low – one key item unavailable; and critically low – more than one key item unavailable.

### 2.5 Data synthesis

The identified SRs were grouped into five major musculoskeletal conditions and the residual musculoskeletal conditions were categorized as other conditions. Musculoskeletal conditions were only eligible for comparative effectiveness analysis if at least three SRs were available for the musculoskeletal conditions. Based on post-intervention time measurements of pain outcome, the impact of acupuncture is categorized as short-term when the outcome is measured within 3 months post-intervention and long-term when the outcome is measured after 3 months post-intervention. Extracted data were tabulated and summarized in the form of an evidence map.

### 2.6 Evidence mapping

Bubble plots were used to depict multidimensional information including:

(a) *X*-axis – acupuncture relative to comparison treatment. The *x*-axis shows the different types of comparison treatments for major musculoskeletal conditions.
(b) *Y*-axis – major musculoskeletal conditions. The *y*-axis shows the specific topic of the major musculoskeletal conditions when available.
(c) Bubble color – effect of acupuncture. Each musculoskeletal condition is plotted in either beneficial or less beneficial for the effect of acupuncture based on meta-analysis findings.
(d) Bubble size – number of included SRs. Each bubble size is proportional to the number of SRs evaluating the pain outcomes associated with acupuncture intervention.

Each condition (*y*-axis) is presented in the form of color (effect of intervention) and size (research volume) across different comparators (*x*-axis).

## 3 Results

### 3.1 Study screening

**FIGURE 1:**
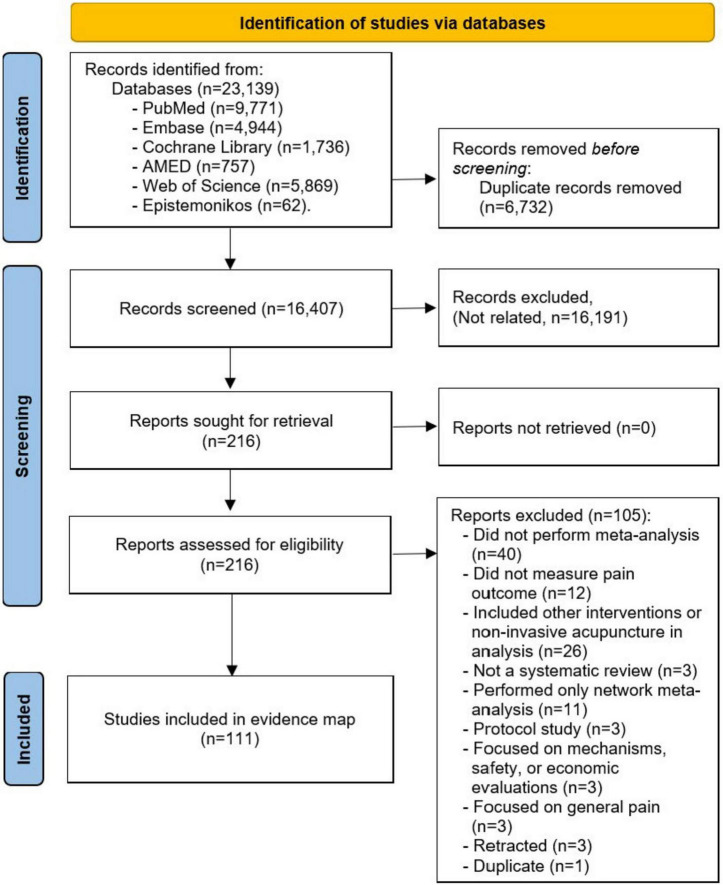
Flowchart outlining the study selection process.

The search identified 16,407 records after duplicates were removed. After reviewing the titles and abstracts, 216 full-text articles were further reviewed for potential inclusion. A total of 111 SRs fulfilled the eligibility criteria and were included in this evidence map. The list of the 105 excluded studies and reasons for exclusion are available in Supplementary Table 2. A flowchart outlining the process of the study selection is detailed in Figure 1.

### 3.2 Study characteristics

Systematic reviews included in this evidence map were published from 1998 to 2024, with the majority published within the last 4 years (64/111, 57.66%). The highest number of SRs originated from China (72/111, 64.9%), followed by South Korea (15/111, 13.5%). Other countries included United States (5/111, 4.5%), United Kingdom (5/111, 4.5%), Canada (4/111, 3.6%), Spain (3/111, 2.7%), Taiwan (3/111, 2.7%), Australia (2/111, 1.8%), Germany (1/111, 0.9%), and Netherlands (1/111, 0.9%).

The number of primary studies included in the SRs ranged from 2 to 77, with a total of 1,776 studies. Twenty-six SRs included less than 10 studies, 64 SRs included 10–20 studies, 9 SRs included 20–30 studies, 8 SRs included 30–40 studies, and 4 SRs included 50 or more studies. The sample size ranged from 84 to 9,650. All of the SRs included RCTs except for one SR which included case-controlled trials. Most of the SRs were non-Cochrane reviews and 8 of the SRs were Cochrane reviews.

All of the SRs included manual acupuncture or electroacupuncture. Seventy-five SRs included more than one type of acupuncture, 16 SRs included all types of acupuncture, 7 SRs included only electroacupuncture, and 13 SRs included only manual acupuncture. Comparators included in SRs involved active comparators, inactive comparators, sham acupuncture, and no intervention. Only 15 SRs reported on the diagnostic criteria for the musculoskeletal conditions included. All SRs employed VAS or NRI as pain outcome measurement. In addition, 69 SRs (62.2%) assessed adverse events and no major adverse events were reported. Forty-two SRs (37.8%) did not report adverse events or relevant data.

The included 111 SRs were categorized into 35 musculoskeletal pain conditions which were further categorized into major musculoskeletal disorders (56/111, 50.5%) and other musculoskeletal disorders (55/111, 49.5%). Major musculoskeletal disorders are categorized according to the Global Burden of Diseases, Injuries, and Risk Factors Study (GBD) (14) which includes low back pain (*n* = 22), osteoarthritis (*n* = 19), neck pain (*n* = 6), rheumatoid arthritis (*n* = 5), and gout (*n* = 4) (Table 1).

**TABLE 1:** Categorization of musculoskeletal conditions.

Musculoskeletal pain category	No. of SRs
**Major musculoskeletal pain**	
Low back pain	22
Osteoarthritis	19
Neck pain	6
Rheumatoid arthritis	5
Gout	4
**Area-specific pain**	
Back pain	22
Knee pain	17
Shoulder pain	9
Neck pain	6
Jaw pain	3
Leg pain	3
Ankle pain	2
Elbow pain	2
Hip pain	2
Pelvic pain	2
Spinal pain	2
Wrist pain	1
**Duration of pain**	
Chronic pain	93
Acute pain	14
**Type of pain**	
Mechanical pain	64
Neuropathic pain	14
Inflammatory pain	14
Central pain	6
Postoperative pain	6
Traumatic pain	4
Functional pain	2
Visceral pain	2

Aside from five specific musculoskeletal condition categories, the residual musculoskeletal disorders of a wide range of acute and chronic conditions are categorized as the sixth category of other musculoskeletal disorders (14). Other musculoskeletal pain in this evidence map includes fibromyalgia (*n* = 6), chemotherapy-induced peripheral neuropathy (*n* = 3), osteoporosis (*n* = 3), postoperative knee arthroplasty (*n* = 3), sciatica (*n* = 3), temporomandibular disorders (*n* = 3), aromatase inhibitor-related arthralgia (*n* = 2), chronic knee pain (*n* = 2), chronic pelvic pain (*n* = 2), chronic spinal pain (*n* = 2), frozen shoulder (*n* = 2), lateral epicondylalgia (*n* = 2), injury-related pain (*n* = 2), postoperative acute orthopedic pain (*n* = 2), post-stroke shoulder pain (*n* = 2), post-stroke shoulder-hand syndrome (*n* = 2), acute ankle sprains (*n* = 1), carpal tunnel syndrome (*n* = 1), chronic ankle instability (*n* = 1), delayed-onset muscle soreness (*n* = 1), diabetic neuropathic pain (*n* = 1), fracture-related pain (*n* = 1), hip pain (*n* = 1), myofascial pain syndrome (*n* = 1), neuropathic pain (*n* = 1), diabetic peripheral neuropathy (*n* = 1), postoperative pain of lumbar disc herniation (*n* = 1), rotator cuff diseases (*n* = 1), shoulder adhesive capsulitis (*n* = 1), and shoulder pain (*n* = 1).

The included musculoskeletal pain conditions were further categorized into area-specific pains: back pain (22/111, 19.8%), knee pain (17/111, 15.3%), shoulder pain (9/111, 8.1%), neck pain (6/111, 5.4%), jaw pain (3/111, 2.7%), leg pain (3/111, 2.7%), ankle pain (2/111, 1.8%), elbow pain (2/111, 1.8%), hip pain (2/111, 1.8%), pelvic pain (2/111, 1.8%), spinal pain (2/111, 1.8%), and wrist pain (1/111, 0.9%).

In terms of pain duration, chronic pain was predominant (70/111, 63.1%), while acute pain was less common (41/111, 36.9%). The most prevalent pain type was mechanical (51/111, 45.7%), followed by neuropathic and inflammatory pain (11/111, 10.2% each), postoperative pain (5/111, 4.5%), traumatic pain (3/111, 2.7%), functional pain (2/111, 1.8%), visceral pain (2/111, 1.8%), and central pain (5/111, 4.5%). Details of the musculoskeletal conditions’ categorization are provided in Supplementary Tables 3, 4.

### 3.3 Impact of acupuncture intervention

The short- and long-term effects of acupuncture on pain outcomes were evaluated across the five major musculoskeletal conditions. Effect directions of each comparison group for acupuncture were reported when available to provide an overall picture of the intervention effects across available evidence. In Table 2, the effect of acupuncture for each major musculoskeletal is listed as either beneficial or less beneficial based on meta-analysis findings. The effect of acupuncture for all included musculoskeletal is listed in Supplementary Table 5.

**TABLE 2:** Effectiveness of acupuncture for major musculoskeletal conditions across comparison groups.

Effect term	Major musculoskeletal conditions	Comparison group	Effect direction
Long-term	Low back pain	AT vs. control	Beneficial
		AT vs. sham AT	Beneficial
		AT vs. usual care	Beneficial
	Osteoarthritis, not specified	AT vs. sham AT	Less beneficial
		AT vs. waitlist	Beneficial
	Knee osteoarthritis	AT vs. sham AT	Beneficial
		AT vs. usual care	Beneficial
	Rheumatoid arthritis	EA vs. sham	Beneficial
	Neck pain	AT vs. sham TENS	Less beneficial
Short-term	Low back pain	AT vs. control	Beneficial
		AT vs. drug	Beneficial
		AT vs. no treatment	Beneficial
		AT vs. sham AT	Beneficial
		AT vs. TENS	Less beneficial
		AT vs. Usual care	Beneficial
		EA vs. drug	Beneficial
		MA vs. sham AT	Beneficial
		WA vs. control	Beneficial
		WA vs. drug	Beneficial
	Osteoarthritis, not specified	AT vs. control	Beneficial
		MA vs. control	Beneficial
		WA vs. control	Beneficial
		WA vs. drug	Beneficial
	Hip osteoarthritis	AT vs. control	Beneficial
		AT vs. drug	Beneficial
		AT vs. sham AT	Less beneficial
	Knee osteoarthritis	AT vs. control	Beneficial
		AT vs. drug	Beneficial
		AT vs. no treatment	Beneficial
		AT vs. sham AT	Beneficial
		AT vs. usual care	Beneficial
		AT vs. waitlist	Beneficial
		EA vs. AT	Beneficial
		EA vs. control	Less beneficial
		EA vs. drug	Less beneficial
		EA vs. sham EA	Beneficial
		MA vs. drug	Less beneficial
	Peripheral joint osteoarthritis	AT vs. sham AT	Beneficial
		MA vs. sham AT	Beneficial
	Neck pain	AT vs. control	Beneficial
		AT vs. sham AT	Beneficial
		EA vs. control	Beneficial
		EA vs. no treatment	Less beneficial
		EA vs. sham EA	Less beneficial
		EA vs. usual care	Beneficial
		MA vs. control	Beneficial
		MA vs. drug	Beneficial
		MA vs. no treatment	Less beneficial
		MA vs. rehabilitation therapy	Beneficial
		MA vs. sham AT	Beneficial
		MA vs. usual care	Beneficial
	Gout	EA vs. drug	Beneficial
		MA vs. control	Beneficial
		WA vs. drug	Beneficial

AT, acupuncture; EA, electro-acupuncture; MA, manual acupuncture; TENS, transcutaneous electrical nerve stimulation; WA, warm acupuncture. Control, no-acupuncture treatment (including physiotherapy, conventional therapy, no intervention, standard treatment, and sham AT); Long-term, more than 3 months post-intervention; short-term, less than 3 months post-intervention.

#### 3.3.1 Short-term effects of acupuncture

As shown in Table 2, the effects of acupuncture show a positive effect across most comparators in major musculoskeletal pain. For low back pain, the included SRs showed a positive effect of acupuncture compared to sham, usual care, drug, and no treatment, but showed an equivalent effect compared to transcutaneous electrical nerve stimulation (TENS) intervention. For osteoarthritis, the comparative effectiveness of acupuncture with no-acupuncture controls showed positive effects in the overall analysis. Yet, it is noted that acupuncture compared to sham for hip osteoarthritis, and electro-acupuncture or manual acupuncture compared to drug for knee osteoarthritis, showed statistically non-significant results. For neck pain, the included SRs reported that acupuncture was more effective than most no-acupuncture controls, but electro-acupuncture was no more effective than no treatment or sham, and similar findings for manual acupuncture compared to no treatment. In the treatment of gouty arthritis, included SRs showed that acupuncture was superior to no-acupuncture controls.

#### 3.3.2 Long-term effects of acupuncture

Based on the findings of meta-analysis, the included SRs showed a positive effect of acupuncture for the treatment of low back pain. For osteoarthritis, it is noted that acupuncture compared to sham showed no better effect but showed a positive effect as compared to other no-acupuncture controls. In the treatment of neck pain, acupuncture compared to sham TENS tended toward an equivalent effect.

### 3.4 Evidence maps

**FIGURE 2:**
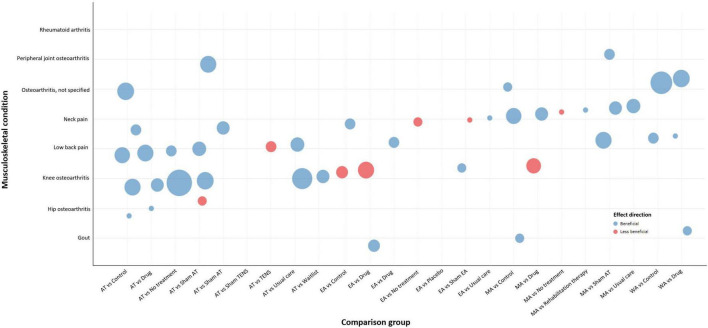
Short-term effect of acupuncture for pain.

**FIGURE 3:**
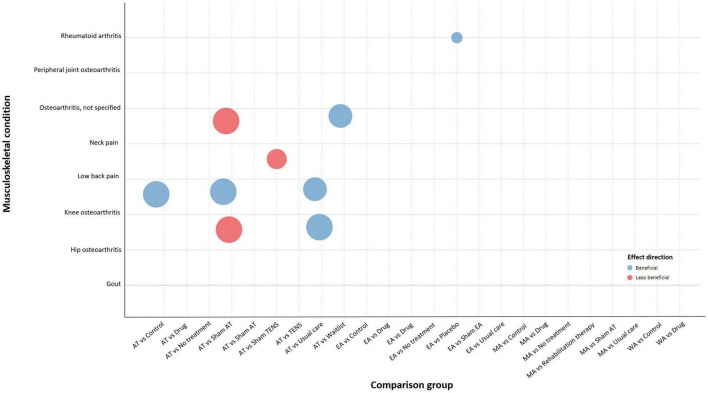
Long-term effect of acupuncture for pain.

The bubble plot summarizes the results for distinct major musculoskeletal conditions relevant to the pain outcome. The bubble plot depicts the treatment effect of acupuncture compared to various active and inactive controls and the estimated research volume based on the number of published SRs. Figure 2 shows the results of short-term acupuncture effect on musculoskeletal pain, and Figure 3 shows the results of long-term acupuncture effect on musculoskeletal pain. Two bubble plots with four depicted dimensions provide a broad visual overview of the evidence.

### 3.5 Methodological quality of SRs

According to AMSTAR 2, 23 SRs were rated “low,” and 88 SRs were rated “critically low.” The least often met criteria were Items 2, 7, and 10. None of the studies (0.0%) fully met the criteria for Item 4. The main reason for the downgrading in Item 4 was the inclusion of content experts in the field for the search strategy used, which was not reported by any of the included SRs. Yet, 81.1% of the included SRs managed to partially meet the criteria Item 2. 93.7% of SRs did not report on the sources of funding in primary studies (Item 10) and 87.4% of SRs did not report the list of excluded studies with reasons for exclusion (Item 7). The details of the AMSTAR 2 scoring were listed in Supplementary Table 6.

## 4 Discussion

### 4.1 Overview of findings

This evidence map included 111 SRs across 2 major musculoskeletal disorder categories and 35 musculoskeletal pain conditions. The most frequently studied musculoskeletal pain was low back pain followed by osteoarthritis. Most meta-analysis findings for major musculoskeletal disorders have shown that acupuncture treatments are beneficial for pain conditions and there was no adverse event evidence showing that acupuncture was least safe. Despite the promising effect, the methodological quality of the included SRs was modest and required further research.

### 4.2 Area of major gaps in findings

The available evidence compiled in this map shows that acupuncture has short-term benefits in relieving pain for musculoskeletal conditions. However, there was a lack of evidence for the long-term effectiveness of acupuncture in treating musculoskeletal pain. Acupuncture treatment periods are generally 4, 8, and 12 weeks (15), and many trials do not have follow-ups to evaluate the long-term effects. Therefore, the evidence is insufficient to support the use of acupuncture for improving pain symptoms for musculoskeletal conditions in the long-term.

Another gap identified was the research volume for other musculoskeletal conditions. Besides the major musculoskeletal conditions identified as the GBD (14), there are relatively less evidence to demonstrate the benefits of acupuncture on other musculoskeletal pain such as carpal tunnel syndrome, shoulder pain and hip pain among others.

### 4.3 Implications for practice and research

Despite the available evidence on acupuncture in treating musculoskeletal pain, most SRs conclude that the evidence is uncertain and warrant more researches to be conducted. Therefore, researchers should prioritize the quality of trials and investigate on topic areas where evidence is still limited. There is a need for more high-quality evidence for health professionals to utilize and recommend the use of acupuncture, and for patients to have confidence in the benefit of acupuncture for their musculoskeletal conditions.

Also, the researchers should avoid producing similar SRs to prevent research waste. In recent years, the number of SRs has increased overwhelmingly (16). There are as many SRs as clinical trials since conducting a clinical trial involves many barriers, such as funding, time-consuming, human capacity, patient recruitment, and ethical issues (17–19). Yet, evidence from SRs are highly dependent on the included primary studies (20–22). Therefore, the research focus should be on increasing the SR evidence quality by improving the quality of clinical trials to address the insufficient high-quality evidence.

In addition, this evidence map highlights the geographic concentration of acupuncture research in Asia, limiting the generalizability of findings. While acupuncture is a global modality, the preponderance of studies from this region reflects local practices. Future research should prioritize geographic diversity to enhance the global applicability of acupuncture and support evidence-based practice.

### 4.4 Limitations

This study has limitations. The first limitation of the evidence map is that we may not have identified all available evidence due to the limited database search. The second limitation is that we did not evaluate the quality or overlapping of primary studies in the included SRs. In this evidence map, we took the meta-analysis findings of the authors of SRs as it is in determining the effect direction of acupuncture. The third limitation is that the definition of control varies from studies to studies. Some SRs did not define control and this somehow limits us to compare the benefits of acupuncture over certain control.

## 5 Conclusion

This evidence map showed that acupuncture has beneficial effects on musculoskeletal pain, especially for major musculoskeletal disorders. The quality of evidence was concerning and future researches should focus on increasing the quality of clinical trials.

## Authors contributions

LA: Conceptualization, Data curation, Formal Analysis, Investigation, Methodology, Writing – original draft, Writing – review and editing. ES: Conceptualization, Formal Analysis, Investigation, Methodology, Writing – original draft, Writing – review and editing. T-YC: Investigation, Methodology, Writing – review and editing. JJ: Investigation, Methodology, Writing – review and editing. BL: Investigation, Methodology, Writing – review and editing. MY: Data curation, Formal Analysis, Visualization, Writing – review and editing. HL: Methodology, Resources, Writing – review and editing. ML: Conceptualization, Funding acquisition, Methodology, Resources, Supervision, Writing – review and editing.

## Data Availability

The original contributions presented in this study are included in this article/Supplementary material, further inquiries can be directed to the corresponding author.
