# Supplementary material for: Effects of acupuncture on musculoskeletal pain: an evidence map
Source: Front Med (Lausanne). 2025 Aug 11;12:1575226. doi: 10.3389/fmed.2025.1575226 (PMC12375662; doi:10.3389/fmed.2025.1575226)
Supplement: Supplementary file 1 [file Table_1.docx]

**Supplemental Table 1. Search strategy**

| **<PubMed>**  (Acupuncture[MH] OR “Acupuncture Points”[MH] OR “Acupuncture Therapy”[MH] OR “Dry Needling”[MH] OR Electroacupuncture[MH] OR “Electric Stimulation Therapy”[MH] OR “Acupuncture, Ear”[MH] OR Auriculotherapy[MH] OR “Bee Venoms”[MH] OR Apitherapy[MH] OR acupuncture*[TW] OR acupoint*[TW] OR needle*[TW] OR needling[TW] OR electroacupuncture[TW] OR electro-acupuncture[TW] OR “electric stimulation therap*”[TIAB] OR pharmacopuncture[TW] OR pharmacoacupuncture[TW] OR “herbal injection”[TIAB] OR “ear point”[TIAB] OR thread-embedding[TIAB] OR needle-embedding[TIAB] OR “embedding therapy”[TIAB] OR “catgut embedding”[TIAB] OR “catgut implantation”[TIAB] OR “bee venom* therapy”[TIAB] OR apitherapy[TIAB] OR “trigger point*”[TIAB] OR acupotom*[TIAB] OR “needle-knife”[TIAB] OR miniscalpel[TIAB]) AND ("pain"[MH] OR "pain management"[MH] OR "back pain"[MH] OR "backache"[MH] OR "bone"[MH] OR "bursitis"[MH] OR "carpal tunnel syndrome"[MH] OR "chronic musculoskeletal pain"[MH] OR "chronic pain"[MH] OR "fibromyalgia"[MH] OR "foot pain"[MH] OR "heel pain"[MH] OR "heel spur"[MH] OR "herniated disc"[MH] OR "hip bursitis"[MH] OR "hip pain"[MH] OR "joint"[MH] OR "knee pain"[MH] OR "leg cramps"[MH] OR "lower back pain"[MH] OR "lumbar pain"[MH] OR "muscle"[MH] OR "muscle relaxers"[MH] OR "muscle spasm"[MH] OR "musculoskeletal disease"[MH] OR "musculoskeletal pain"[MH] OR "myalgia"[MH] OR "neck pain"[MH] OR "osteoarthritis"[MH] OR "piriformis syndrome"[MH] OR "rheumatoid arthritis"[MH] OR "sciatic nerve pain"[MH] OR "sciatica"[MH] OR "shoulder pain"[MH] OR "analgesia"[TW] OR "pain"[TIAB] OR "pain management"[TIAB] OR "pain"[TW] OR "pain management"[TW] OR "back pain"[TIAB] OR "back pain"[TW] OR "bone"[TW] OR "bursitis"[TW] OR "carpal tunnel syndrome"[TIAB] OR "carpal tunnel syndrome"[TW] OR "chronic musculoskeletal pain"[TIAB] OR "chronic pain"[TIAB] OR "fibromyalgia"[TIAB] OR "fibromyalgia"[TW] OR "foot pain"[TW] OR "heel pain"[TW] OR "heel spur"[TW] OR "herniated disc"[TIAB] OR "herniated disc"[TW] OR "hip bursitis"[TW] OR "hip pain"[TW] OR "joint"[TIAB] OR "knee pain"[TIAB] OR "knee pain"[TW] OR "leg cramps"[TW] OR "lower back pain"[TIAB] OR "lumbar pain"[TIAB] OR "lower back pain"[TW] OR "lumbar pain"[TW] OR "muscle"[TW] OR "muscle relaxers"[TW] OR "muscle spasm"[TW] OR "musculoskeletal disease"[TIAB] OR "musculoskeletal pain"[TIAB] OR "musculoskeletal disease"[TW] OR "musculoskeletal pain"[TW] OR "myalgia"[TW] OR "neck pain"[TW] OR "osteoarthritis"[TW] OR "piriformis syndrome"[TW] OR "rheumatoid arthritis"[TW] OR "sciatic nerve pain"[TW] OR "sciatica"[TW] OR "shoulder pain"[TW]) AND (“Meta-Analysis as Topic”[MH] OR “Review Literature as Topic”[MH] OR “Systematic Reviews as Topic”[MH] OR “Systematic Review"[PT] OR [Meta-Analysis[PT]](https://www.ncbi.nlm.nih.gov/mesh/68017418) OR “meta analy*”[TIAB] OR review*[TIAB] OR meta-analysis[TIAB] OR meta-review*[TIAB] OR “systematic map”[TIAB]) |
| --- |

**Supplemental Table 2. List of exclusion with reasons**

| First author | Year | Reasons |
| --- | --- | --- |
| Almutairi | 2022 | Performed only network meta-analysis |
| Bae | 2015 | Did not perform meta-analysis |
| Baroncini | 2022 | Included other interventions or non-invasive acupuncture in analysis |
| Berger | 2021 | Did not perform meta-analysis |
| Berger | 2021 | Not a systematic review |
| Cao | 2013 | Included other interventions or non-invasive acupuncture in analysis |
| Cen | 2020 | Performed only network meta-analysis |
| Chang | 2021 | Included other interventions or non-invasive acupuncture in analysis |
| Chau | 2018 | Did not perform meta-analysis |
| Chen | 2017 | Included other interventions or non-invasive acupuncture in analysis |
| Chen | 2022 | Not a systematic review |
| Chen | 2012 | Did not measure pain outcome |
| Chen | 2019 | Included other interventions or non-invasive acupuncture in analysis |
| Chien | 2019 | Included other interventions or non-invasive acupuncture in analysis |
| Choi | 2018 | Did not measure pain outcome |
| Chou | 2018 | Focused on mechanisms, safety, or economic evaluations |
| Clark | 2012 | Did not perform meta-analysis |
| Corbett | 2013 | Performed only network meta-analysis |
| Cox | 2016 | Did not perform meta-analysis |
| Daya | 2007 | Did not perform meta-analysis |
| Dimitrova | 2017 | Did not measure pain outcome |
| Ee | 2008 | Did not perform meta-analysis |
| Ernst | 1997 | Did not perform meta-analysis |
| Ernst | 1999 | Did not perform meta-analysis |
| Ernst | 2002 | Duplicate with Ernst 1998 (same results published in German) |
| Ezzo | 2001 | Did not perform meta-analysis |
| Fan | 2024 | Did not perform meta-analysis |
| Farag | 2020 | Included other interventions or non-invasive acupuncture in analysis |
| Fernandes | 2017 | Did not perform meta-analysis |
| Gadau | 2014 | Did not perform meta-analysis |
| Green | 2002 | Did not measure pain outcome |
| Guimaraes | 2023 | Included other interventions or non-invasive acupuncture in analysis |
| Ha | 2022 | Did not perform meta-analysis |
| Han | 2022 | Included other interventions or non-invasive acupuncture in analysis |
| Hou | 2020 | Not a systematic review |
| Huang | 2019 | Did not measure pain outcome |
| Huang | 2022 | Performed only network meta-analysis |
| Huh | 2021 | Did not perform meta-analysis |
| Hutchinson | 2012 | Did not perform meta-analysis |
| Jin | 2021 | Did not measure pain outcome |
| Jung | 2011 | Included other interventions or non-invasive acupuncture in analysis |
| Kim | 2013 | Included other interventions or non-invasive acupuncture in analysis |
| Ko | 2020 | Did not perform meta-analysis |
| La Touche | 2010 | Did not perform meta-analysis |
| Lam | 2013 | Included other interventions or non-invasive acupuncture in analysis |
| Lee | 2012 | Did not perform meta-analysis |
| Lenoir | 2020 | Focused on general pain |
| Lewis | 2015 | Included other interventions or non-invasive acupuncture in analysis |
| Li | 2018 | Performed only network meta-analysis |
| Li | 2022 | Included other interventions or non-invasive acupuncture in analysis |
| Li | 2019 | Did not perform meta-analysis |
| Li | 2016 | Included other interventions or non-invasive acupuncture in analysis |
| Lim | 2018 | Did not perform meta-analysis |
| Lin | 2012 | Included other interventions or non-invasive acupuncture in analysis |
| Lin | 2016 | Included other interventions or non-invasive acupuncture in analysis |
| Liu | 2019 | Included other interventions or non-invasive acupuncture in analysis |
| Liu | 2021 | Performed only network meta-analysis |
| Liu | 2021 | Did not measure pain outcome |
| Liu | 2020 | Did not measure pain outcome |
| Lu | 2022 | Included other interventions or non-invasive acupuncture in analysis |
| Ma | 2023 | Included other interventions or non-invasive acupuncture in analysis |
| Mayhew | 2007 | Did not perform meta-analysis |
| McDonald | 2023 | Did not perform meta-analysis |
| Moon | 2014 | Did not perform meta-analysis |
| Nash | 2019 | Did not perform meta-analysis |
| Pai | 2023 | Did not perform meta-analysis |
| Park | 2013 | Did not measure pain outcome |
| Seca | 2019 | Did not perform meta-analysis |
| Selfe | 2008 | Did not perform meta-analysis |
| Shi | 2021 | Included other interventions or non-invasive acupuncture in analysis |
| Sim | 2011 | Included other interventions or non-invasive acupuncture in analysis |
| Sung | 2018 | Did not measure pain outcome |
| Sung | 2021 | Did not perform meta-analysis |
| Tang | 2015 | Did not measure pain outcome |
| Tian | 2022 | Retracted |
| Trigkilidas | 2010 | Did not perform meta-analysis |
| Trinh | 2016 | Retracted |
| Trinh | 2004 | Did not perform meta-analysis |
| Trinh | 2022 | Did not perform meta-analysis |
| Trinh | 2021 | Did not perform meta-analysis |
| Tulder | 2000 | Did not perform meta-analysis |
| Turner | 2013 | Did not perform meta-analysis |
| Wan | 2022 | Included other interventions or non-invasive acupuncture in analysis |
| Wang | 2008 | Did not perform meta-analysis |
| Wang | 2016 | Did not measure pain outcome |
| Wang | 2020 | Protocol study |
| Wang | 2021 | Performed only network meta-analysis |
| Wang | 2016 | Included other interventions or non-invasive acupuncture in analysis |
| Wang | 2022 | Performed only network meta-analysis |
| White | 1999 | Did not perform meta-analysis |
| Wu | 2020 | Did not perform meta-analysis |
| Xu | 2013 | Did not perform meta-analysis |
| Yang | 2018 | Did not measure pain outcome |
| Ye | 2022 | Focused on general pain |
| Yuan | 2016 | Focused on general pain |
| Yuan | 2008 | Did not perform meta-analysis |
| Yuan | 2008 | Did not perform meta-analysis |
| Zhang | 2019 | Protocol study |
| Zhang | 2015 | Included other interventions or non-invasive acupuncture in analysis |
| Zhao | 2022 | Performed only network meta-analysis |
| Zhu | 2014 | Included other interventions or non-invasive acupuncture in analysis |
| Zuo | 2019 | Protocol study |

**Supplemental Table 3. Categorization of chronic and acute pain**

| Pain category | Condition | No. of SRs |
| --- | --- | --- |
| Chronic pain | Low back pain | 22 |
|  | Osteoarthritis | 19 |
|  | Neck pain | 6 |
|  | Rheumatoid arthritis | 5 |
|  | Fibromyalgia | 6 |
|  | Chemotherapy-induced peripheral neuropathy | 3 |
|  | Osteoporosis | 3 |
|  | Postoperative knee arthroplasty | 3 |
|  | Sciatica | 3 |
|  | Temporomandibular disorders | 3 |
|  | Aromatase inhibitor-related arthralgia | 2 |
|  | Chronic knee pain | 2 |
|  | Chronic pelvic pain | 2 |
|  | Chronic spinal pain | 2 |
|  | Frozen shoulder | 2 |
|  | Lateral epicondylalgia | 2 |
|  | Injury-related pain | 2 |
|  | Postoperative acute orthopedic pain | 2 |
|  | Post-stroke shoulder pain | 2 |
|  | Post-stroke shoulder-hand syndrome | 2 |
| Acute pain | Acute ankle sprains | 1 |
|  | Carpal tunnel syndrome | 1 |
|  | Chronic ankle instability | 1 |
|  | Delayed-onset muscle soreness | 1 |
|  | Diabetic neuropathic pain | 1 |
|  | Fracture-related pain | 1 |
|  | Hip pain | 1 |
|  | Myofascial pain syndrome | 1 |
|  | Neuropathic pain (general) | 1 |
|  | Diabetic peripheral neuropathy | 1 |
|  | Postoperative pain of lumbar disc herniation | 1 |
|  | Rotator cuff diseases | 1 |
|  | Shoulder adhesive capsulitis | 1 |
|  | Shoulder pain | 1 |

**Supplemental Table 4. Categorization of pain types**

| Pain type | Condition | No. of SRs |
| --- | --- | --- |
| Neuropathic pain | Chemotherapy-induced peripheral neuropathy | 3 |
|  | Post-stroke shoulder pain | 2 |
|  | Post-stroke shoulder-hand syndrome | 2 |
|  | Sciatica | 3 |
|  | Carpal tunnel syndrome | 1 |
|  | Diabetic neuropathic pain | 1 |
|  | Diabetic peripheral neuropathy | 1 |
|  | Neuropathic pain | 1 |
| Mechanical pain | Low back pain | 22 |
|  | Osteoarthritis | 19 |
|  | Neck pain | 6 |
|  | Postoperative knee arthroplasty | 3 |
|  | Chronic knee pain | 2 |
|  | Frozen shoulder | 2 |
|  | Lateral epicondylalgia | 2 |
|  | Chronic spinal pain | 2 |
|  | Injury-related pain | 2 |
|  | Rotator cuff diseases | 1 |
|  | Shoulder adhesive capsulitis | 1 |
|  | Hip pain | 1 |
|  | Shoulder pain | 1 |
| Inflammatory pain | Rheumatoid arthritis | 5 |
|  | Gout | 4 |
|  | Chronic knee pain | 2 |
|  | Chronic spinal pain | 2 |
|  | Myofascial pain syndrome | 1 |
| Postoperative pain | Postoperative knee arthroplasty | 3 |
|  | Postoperative acute orthopedic pain | 2 |
|  | Postoperative pain of lumbar disc herniation | 1 |
| Traumatic pain | Injury-related pain | 2 |
|  | Acute ankle sprains | 1 |
|  | Fracture-related pain | 1 |
| Functional pain | Aromatase inhibitor-related arthralgia | 2 |
| Visceral pain | Chronic pelvic pain | 2 |
| Central pain | Fibromyalgia | 6 |

* Some conditions may fall under either pain category (mechanical or inflammatory), leading to overlaps between the categories.

**Supplemental Table 5. Effect of acupuncture for all included musculoskeletal conditons**

| Period | Conditions | Comparison group | Effectiveness |
| --- | --- | --- | --- |
| Long term | Fibromyalgia | AT vs sham AT | Beneficial |
|  |  | MA vs Sham AT | Less beneficial |
|  | Knee pain | AT vs Sham AT | Beneficial |
|  | Osteoporosis | EA vs Drug | Beneficial |
|  |  | WA vs Drug | Beneficial |
|  | Acute ankle sprains | AT vs Drug | Beneficial |
|  | Fibromyalgia | AT vs Sham AT | Beneficial |
|  |  | EA vs Sham EA | Less beneficial |
|  | Fracture-related pain | AT vs Drug | Beneficial |
|  | Frozen shoulder | EA vs Control | Beneficial |
|  | Knee osteoarthritis | AT vs Sham AT | Beneficial |
|  |  | AT vs Usual care | Beneficial |
|  | Lateral epicondylalgia | MA vs Control | Beneficial |
|  |  | MA vs Sham AT | Less beneficial |
|  | Low back pain | AT vs Control | Beneficial |
|  |  | AT vs Sham AT | Beneficial |
|  |  | AT vs Usual care | Beneficial |
|  | Neck Pain | AT vs Sham TENS | Beneficial |
|  | Osteoarthritis, not specified | AT vs Sham AT | Less beneficial |
|  |  | AT vs Waitlist | Beneficial |
|  | Rheumatoid arthritis | EA vs Sham | Beneficial |
|  | Spinal pain | AT vs Control | Beneficial |
|  |  | AT vs Sham AT | Beneficial |
| Short term | Acute ankle sprains | AT vs Control | Beneficial |
|  | Aromatase inhibitor-related arthralgia | AT vs Drug | Beneficial |
|  |  | AT vs Sham AT | Less beneficial |
|  |  | AT vs Waitlist | Beneficial |
|  | Carpal tunnel syndrome | AT vs Drug | Beneficial |
|  | Cervical spondylosis | AT vs Control | Beneficial |
|  | Cervical spondylotic radiculopathy | MA vs Control | Less beneficial |
|  | Chronic ankle instability | AT vs Control | Beneficial |
|  | Delayed-onset muscle soreness | AT vs sham AT | Beneficial |
|  | Diabetic peripheral neuropathy | AT vs Drug | Beneficial |
|  | Fibromyalgia | AT vs Control | Beneficial |
|  |  | AT vs Drug | Beneficial |
|  |  | AT vs Sham AT | Beneficial |
|  |  | EA vs Drug | Beneficial |
|  |  | EA vs Sham EA | Beneficial |
|  | Fracture-related pain | AT vs Drug | Beneficial |
|  | Frozen shoulder | MA vs Drug | Beneficial |
|  | Gout | EA vs Drug | Beneficial |
|  |  | MA vs Control | Beneficial |
|  |  | WA vs Drug | Beneficial |
|  | Hip osteoarthritis | AT vs Control | Beneficial |
|  |  | AT vs Drug | Beneficial |
|  |  | AT vs Sham AT | Less beneficial |
|  | Hip pain | AT vs Control | Beneficial |
|  | Knee osteoarthritis | AT vs Control | Beneficial |
|  |  | AT vs Drug | Beneficial |
|  |  | AT vs No treatment | Beneficial |
|  |  | AT vs Sham AT | Beneficial |
|  |  | AT vs Usual care | Beneficial |
|  |  | AT vs Waitlist | Beneficial |
|  |  | EA vs AT | Beneficial |
|  |  | EA vs Control | Less beneficial |
|  |  | EA vs Drug | Less beneficial |
|  |  | EA vs Sham EA | Beneficial |
|  |  | MA vs Drug | Less beneficial |
|  | Knee pain | AT vs No treatment | Beneficial |
|  | Lateral epicondylalgia | AT vs Control | Beneficial |
|  |  | AT vs Drug | Beneficial |
|  |  | AT vs Sham AT | Beneficial |
|  |  | EA vs Control | Less beneficial |
|  |  | MA vs Control | Less beneficial |
|  |  | MA vs Sham AT | Less beneficial |
|  | Low back fasciitis | AT vs Drug | Less beneficial |
|  | Low back pain | AT vs Control | Beneficial |
|  |  | AT vs Drug | Beneficial |
|  |  | AT vs No treatment | Beneficial |
|  |  | AT vs Sham AT | Beneficial |
|  |  | AT vs TENS | Less beneficial |
|  |  | AT vs Usual care | Beneficial |
|  |  | EA vs Drug | Beneficial |
|  |  | MA vs Sham AT | Beneficial |
|  | Lumbar disc herniation | AT vs Control | Beneficial |
|  |  | WA vs Control | Beneficial |
|  |  | WA vs Drug | Beneficial |
|  | Neck pain | AT vs Control | Beneficial |
|  |  | AT vs Sham AT | Beneficial |
|  |  | EA vs Active control | Beneficial |
|  |  | EA vs Control | Beneficial |
|  |  | EA vs No treatment | Less beneficial |
|  |  | EA vs Sham EA | Less beneficial |
|  |  | EA vs Usual care | Beneficial |
|  |  | MA vs Control | Beneficial |
|  |  | MA vs Drug | Beneficial |
|  |  | MA vs No treatment | Less beneficial |
|  |  | MA vs Rehabilitation therapy | Beneficial |
|  |  | MA vs Sham AT | Beneficial |
|  |  | MA vs Usual care | Beneficial |
|  | Neuropathic pain | AT vs Control | Beneficial |
|  |  | AT vs Drug | Beneficial |
|  | Osteoarthritis | AT vs Control | Beneficial |
|  |  | MA vs Control | Beneficial |
|  |  | WA vs Control | Beneficial |
|  |  | WA vs Drug | Beneficial |
|  | Pelvic pain | AT vs Control | Beneficial |
|  |  | EA vs Drug | Beneficial |
|  |  | MA vs Drug | Beneficial |
|  | Peripheral joint osteoarthritis | AT vs Sham AT | Beneficial |
|  |  | MA vs Sham AT | Beneficial |
|  | Peripheral neuropathy | AT vs Control | Beneficial |
|  |  | MA vs Sham AT | Beneficial |
|  | Postoperative acute orthopedic pain | AT vs Control | Beneficial |
|  |  | AT vs Usual care | Beneficial |
|  | Postoperative knee arthroplasty | AT vs Sham AT | Beneficial |
|  |  | EA vs Control | Beneficial |
|  | Postoperative pain of lumbar disc herniation | AT vs Drug | Beneficial |
|  |  | AT vs Rehabilitation therapy | Beneficial |
|  | Post-stroke shoulder-hand syndrome | AT vs Control | Beneficial |
|  |  | EA vs Control | Beneficial |
|  | Rheumatoid arthritis | AT vs Sham AT | Less beneficial |
|  |  | EA vs Control | Beneficial |
|  |  | EA vs Placebo | Beneficial |
|  |  | MA vs Sham AT | Less beneficial |
|  |  | WA vs Drug | Less beneficial |
|  | Rotator cuff diseases | AT vs Rehabilitation therapy | Beneficial |
|  | Sciatica | AT vs Drug | Beneficial |
|  |  | AT vs Sham AT | Beneficial |
|  | Shoulder adhesive capsulitis | AT vs Control | Beneficial |
|  |  | AT vs Sham AT | Less beneficial |
|  | Shoulder pain | AT vs Control | Less beneficial |
|  |  | AT vs Drug | Less beneficial |
|  |  | AT vs Rehabilitation therapy | Less beneficial |
|  |  | AT vs Sham AT | Less beneficial |
|  |  | EA vs Drug | Beneficial |
|  |  | EA vs Rehabilitation therapy | Less beneficial |
|  |  | MA vs Control | Beneficial |
|  |  | MA vs Rehabilitation therapy | Less beneficial |
|  | Spinal cord injury | AT vs Drug | Less beneficial |
|  | Spinal pain | AT vs Control | Beneficial |
|  |  | AT vs Sham AT | Beneficial |
|  | Temporomandibular disorders | AT vs Control | Beneficial |
|  |  | AT vs Sham AT | Beneficial |
|  | Whiplash injury | AT vs Control | Beneficial |
|  |  | EA vs Control | Less beneficial |

**Supplemental Table 6. Systematic reviews scoring for AMSTAR 2 items**

| Item | AMSTAR 2 items | Yes, n (%) | No, n (%) | Partial yes, n (%) |
| --- | --- | --- | --- | --- |
| 1 | Included PICO | 60 (54.1) | 51 (45.9) | - |
| 2* | Review methods were established prior to the conduct of the review | 18 (16.2) | 61 (55.0) | 32 (28.8) |
| 3 | Explain selection of the study designs | 56 (50.5) | 55 (49.5) | - |
| 4* | Use a comprehensive search strategy | 0 (0.0) | 21 (18.9) | 90 (81.1) |
| 5 | Perform study selection in duplicate | 87 (78.4) | 24 (21.6) | - |
| 6 | Perform data extraction in duplicate | 85 (76.6) | 26 (23.4) | - |
| 7* | Provide a list of excluded studies with justification | 13 (11.7) | 97 (87.4) | 1 (0.9) |
| 8 | Describe the included studies in detail | 34 (30.6) | 8 (7.2) | 69 (62.2) |
| 9* | Use a satisfactory technique for assessing the RoB | 79 (71.2) | 13 (11.7) | 19 (17.1) |
| 10 | Report on the sources of funding in primary studies | 7 (6.3) | 104 (93.7) | - |
| 11* | Use appropriate methods for pooling results | 69 (62.2) | 42 (37.8) | - |
| 12 | Assess the potential impact of RoB in meta-analysis results/other evidence synthesis | 59 (53.2) | 51 (45.9) | 1 (0.9) |
| 13* | Account for RoB in individual studies when interpreting/discussing results | 66 (59.5) | 45 (40.5) | - |
| 14 | Discussion of any heterogeneity observed in the results | 84 (75.7) | 27 (24.3) | - |
| 15* | Investigation of publication bias | 61 (55.0) | 50 (45.0) | - |
| 16 | Report conflict of interest/funding | 94 (84.7) | 17 (15.3) | - |

PICO, population, intervention, comparator, and outcome; RoB, risk of bias; *, critical domain
